# Crohn’s Disease-Like Features in a Patient With IgE and Selective IgG1 and IgG3 Deficiency

**DOI:** 10.7759/cureus.34655

**Published:** 2023-02-05

**Authors:** Ioannis Drygiannakis, Eirini Theodoraki, Maria Tsafaridou, Ioannis Koutroubakis

**Affiliations:** 1 Department of Gastroenterology, University General Hospital of Heraklion, Heraklion, GRC

**Keywords:** crohn’s disease, primary immune deficiency, primary immunodeficiency, ige deficiency, selective igg deficiency, crohn’s-like disease

## Abstract

We report a 19-year-old male with congenital, combined deficiency of immunoglobulin (Ig) E and 2/4 subclasses of IgG (G1, G3) and chronic diarrhea. He presented at six years of age with chronic recurrent diarrhea responsive to immunoglobulin treatment. Initially, it was considered of infectious origin. However, at the age of 14 years, ileocolonoscopy and magnetic resonance enterography (MRE) were performed, and they showed a mild, limited, non-specific, terminal ileitis with increased eosinophil count on histology. A diagnosis of possible eosinophilic gastroenteritis was made, and budesonide was administered with temporary relief. However, at the age of 19 years, repeat ileocolonoscopy showed multiple ulcers in the terminal ileum and aphthous ulcers in the cecum, and repeat MRE demonstrated extensive ileal involvement. Esophagogastroduodenoscopy demonstrated the involvement of the upper GI tract with aphthous ulcers. Subsequently, gastric, ileal, and colonic biopsies revealed Ziehl-Neelsen-negative, non-caseating granulomas. We hereby report the first case of IgE and selective IgG1 and IgG3 deficiency complicated with Crohn’s disease-like extensive GI involvement.

## Introduction

Hypogammaglobulinemias are heterogeneous diseases that present in immune disorders, such as selective immunoglobulin (Ig) A deficiency (SIGAD) [1], primary human immune deficiency (PHID), and common variable immunodeficiency (CVID). CVID is an umbrella diagnosis for many heterogeneous conditions of impaired humoral immunity. However, it invariably includes a ≥ 2 SDs decrease of all IgG subclasses (1-4) in addition to a decrease of IgA or IgM classes. CVID has an enteropathy component that cannot be easily distinguished from Crohn’s disease (CD) since both can have identical clinical presentation and pathology [2]. Several cases of primary hypogammaglobulinemia and GI manifestations mimicking CD have been reported [2,3]. In a recent, large case series study of 27 patients [3] with primary hypogammaglobulinemia and inflammatory bowel disease (IBD)-like disease, 18 had CVID and three had SIGAD. Only one had selective IgG subclass deficiency, a disorder with 1-2 out of 4 IgG subclasses being below the lower normal limit (LNL), together with IgA deficiency [3]. None of those patients had upper GI tract involvement or IgE deficiency [3]. After a review of the literature, we report the first patient with a combined deficiency of IgE and subclasses 1 and 3 of IgG (thus selective IgG deficiency), complicated with a CD-like disease of both the upper and the lower GI tract.

## Case presentation

Symptoms and history

A 19-year-old male smoker (4 pack years) was referred for intermittent episodes of diarrhea lasting one to a few months since he was six years old. At presentation, he reported approximately 10 watery bowel movements per day for the last two months, often awakening him at night. He also reported urgency and diffuse, crampy abdominal pain, relieved upon defecation and odynophagia. He was born by cesarean section and, as a neonate, was admitted to the pediatric ICU with bacterial meningitis by Staphylococcus epidermidis, responsive to the standard regimen. At the age of one year, he was diagnosed with Hirschsprung's disease, which was repaired by the Duhamel procedure. Moreover, in the first years of his life, he had recurrent infections (bronchiolitis, sinusitis, pharyngitis, otitis media, and, to a less extent, acute gastroenteritis), and low serum levels of total IgG and IgE were found. Subsequent measurement of IgG (1-4) subclasses showed selective IgG1 and IgG3 deficiency. A diagnosis of selective IgG, with subclasses IgG1 and IgG3 affected, and IgE (IgG1&G3&E) deficiency was made. He was started on IV IgG infusions, resulting in a reduced incidence of infections and a normal growth curve. Serum Igs were routinely measured immediately before and after infusions and consistently verified decreased levels before, as opposed to normal ones after. His medical history was also crucial for Hashimoto's thyroiditis and rare events of aphthous stomatitis. Most episodes of diarrhea were self-limited or successfully treated with antibiotics empirically, but the bacterial infection was only proven once with Campylobacter jejuni resistant to ciprofloxacin in fecal cultures. Further, serology for viruses was consistently negative for IgM but demonstrated IgG for cytomegalovirus (CMV), Epstein-Barr virus (EBV), Parvovirus, varicella zoster virus (VZV), herpes simplex virus-1 (HSV-1). However, such serology might be due to IV Ig treatment. Of note, he was not compliant with his treatment since the age of 13.

Diagnosis

An ileocolonoscopy at the age of 14 years showed mild, non-specific ileitis with an eosinophilic infiltrate in biopsies. Due to a possible diagnosis of eosinophilic gastroenteritis, he received episodic treatment with budesonide providing temporary relief.

At the presentation, he admitted having abandoned treatment with IgG for the previous year. He reported low-grade fever but, otherwise, had a normal physical examination. However, he had increased C-reactive protein, erythrocyte sedimentation rate, and WBCs (Table 1). IgG1 and IgG3, as well as total IgG, were below the normal range (Table 1). Serology for anti-cytomegalovirus IgM antibodies, stool culture and microscopy for parasites from three different samples, and *Clostridium difficile* toxins A and B from a single sample were all negative for pathogens (Table 1).

**Table 1:** Laboratory tests at presentation CRP: C-reactive protein; ESR: Erythrocyte sedimentation rate; Ig: Immunoglobulin; CMV: Cytomegalovirus; x3: From three different samples; *C.*: *Clostridium.*

	Value	Normal range
Peripheral venous blood		
CRP (mg/dl)	1.75	<0.5
ESR (mm)	33	1-25
WBC (K/ul)	11.2	3.8-10.5
IgG (mg/dl)	615	701-1600
IgG1	473	490-1140
IgG3	18	20-110
CMV IgM	Negative	Negative
Blood culture x3	Sterile	Sterile
Stool		
Culture x3	Normal flora	Normal flora
Microscopy for parasites x3	Negative	Negative
*C. difficile* toxins A, B	Negative	Negative

**Figure 1:**
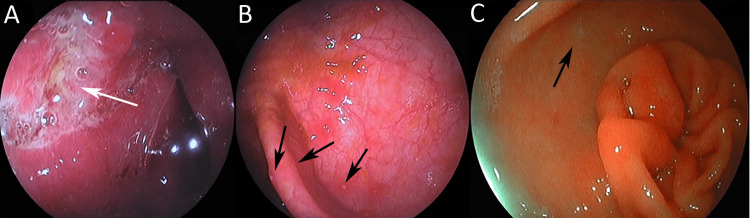
Endoscopy. A) Terminal ileum: no visible villi, erythematous mucosa, or ulcer (white arrow). B) Cecum: aphthous ulcers (black arrows) within normal mucosa. C) Stomach antrum: aphthous ulcer (black arrow) within normal mucosa.

Repeat ileocolonoscopy showed multiple ulcers of irregular shape and variable size on the terminal ileum (Figure 1A) and a few cecal aphthous ulcers (Figure 1B). Ileal and cecal biopsies showed an inflammatory infiltrate of lymphocytes, plasma cells, eosinophils and neutrophils, unevenly distributed in the submucosa, and Ziehl-Neelsen-negative, non-caseating granulomas. Cumulatively, findings of endoscopy and pathology were highly similar to CD [2,3]. Magnetic resonance enterography (MRE) showed extensive ileal involvement with thickening and layering patterns of contrast enhancement in the distal 65 cm of the ileum. Upper GI endoscopy showed esophageal ulcerations and aphthous gastric (Figure 1C) and duodenal ulcers. Biopsies from the stomach and the duodenum showed similar granulomatous inflammation. Based on these findings, a diagnosis of an extensive CD-like disease was made.

Treatment

Initial treatment with antibiotics (ciprofloxacin and metronidazole) had no effect. He was set back to his regular IgG IV infusion schedule. Per os (po), esomeprazole 40 mg once daily and sucralfate 10 ml twice daily were also started.

Outcome

Partial clinical and biochemical improvement was noticed 15 weeks later, and despite his adherence to the IgG infusion schedule, both diarrhea and abnormal biomarkers recurred. Infections were once more ruled out, and po methylprednisolone at 0.75 mg/kg/d was started. He had an inadequate response and was shortly after started on subcutaneous adalimumab (160, 80, and 40 mg thereafter every two weeks). Partial response to adalimumab faded rapidly, parallel to corticosteroids' tapering. After three months, he was switched to IV infliximab (5 mg/kg at 0, 2, 6, and every 8 weeks thereafter) with a complete clinical and biochemical response.

## Discussion

This is the first report of CD-like disease with IgE and selective IgG 1 and 3 deficiency. One patient with CD-like disease and IgG subclass deficiency has been reported, but unlike our patient, he was IgE-competent, had IgA deficiency, and had no upper GI tract involvement [3]. On the other hand, isolated IgG4 deficiency is as frequent as 20% in IBD patients and defines a more severe phenotype [4-7]. No patient with CD and isolated IgE deficiency has been reported so far. On the contrary, CD patients with active disease usually have increased IgE [8]. There was a significant delay in the diagnosis despite the presence of relevant symptoms and abnormal biomarkers for 13 years. This was due to the patient's hesitation to repeat GI endoscopy in adolescence and to IgG infusions which stand as an effective treatment for CD [3,9-12]. The co-existence of CD and selective IgG subclass deficiency could be coincident, and a strong argument for this is the independent clinical course of GI disease and infections at other sites as a response to IV IgG. However, genotyping of the immunoglobulin hypervariable region genes in naïve B cells showed a differential representation of variants in CD compared to healthy controls. This is a hint that IgG subclasses are correlated to the pathogenesis of CD [8]. IgG subclass deficiency may actually be more frequent but underestimated in clinical medicine, as the measurement of IgG subclasses in CD is not routine. Regarding the treatment of primary hypogammaglobulinemia with CD-like features, the classic therapeutic algorithm of CD is used, but additional caution against the use of immunosuppressive agents is suggested. In cases of severe enteropathy, there are a few reports on the successful use of biologics (infliximab, adalimumab, vedolizumab, ustekinumab), like in the reported case. However, long-term safety and treatment duration are not yet clear [3].

## Conclusions

In conclusion, this unique case highlights that, in the presence of a mild congenital deficiency of humoral immunity, physicians should be alert not only on GI infections but on alternative diagnoses, including CD-like disease, and appropriately treat them.
